# Pre-Analytical Parameters Affecting Vascular Endothelial Growth Factor Measurement in Plasma: Identifying Confounders

**DOI:** 10.1371/journal.pone.0145375

**Published:** 2016-01-05

**Authors:** Johanna M. Walz, Daniel Boehringer, Heidrun L. Deissler, Lothar Faerber, Jens C. Goepfert, Peter Heiduschka, Susannah M. Kleeberger, Alexa Klettner, Tim U. Krohne, Nicole Schneiderhan-Marra, Focke Ziemssen, Andreas Stahl

**Affiliations:** 1 Eye Center, University of Freiburg, Freiburg, Germany; 2 Department of Pharmacology and Toxicology, University of Regensburg, Regensburg, Germany; 3 Department of Ophthalmology, University of Ulm, Ulm, Germany; 4 Novartis Pharma AG, Nuremberg, Germany; 5 NMI Natural and Medical Sciences Institute at the University of Tuebingen, Reutlingen, Germany; 6 Department of Ophthalmology, University of Muenster, Muenster, Germany; 7 Department of Ophthalmology, University of Bonn, Bonn, Germany; 8 Department of Ophthalmology, University of Kiel, University medical center, Kiel, Germany; 9 Department for Ophthalmology, Eberhard-Karl University Tuebingen, Tuebingen, Germany; University of Glasgow, UNITED KINGDOM

## Abstract

**Background:**

Vascular endothelial growth factor-A (VEGF-A) is intensively investigated in various medical fields. However, comparing VEGF-A measurements is difficult because sample acquisition and pre-analytic procedures differ between studies. We therefore investigated which variables act as confounders of VEGF-A measurements.

**Methods:**

Following a standardized protocol, blood was taken at three clinical sites from six healthy participants (one male and one female participant at each center) twice one week apart. The following pre-analytical parameters were varied in order to analyze their impact on VEGF-A measurements: analyzing center, anticoagulant (EDTA vs. PECT / CTAD), cannula (butterfly vs. neonatal), type of centrifuge (swing-out vs. fixed-angle), time before and after centrifugation, filling level (completely filled vs. half-filled tubes) and analyzing method (ELISA vs. multiplex bead array). Additionally, intrapersonal variations over time and sex differences were explored. Statistical analysis was performed using a linear regression model.

**Results:**

The following parameters were identified as statistically significant independent confounders of VEGF-A measurements: analyzing center, anticoagulant, centrifuge, analyzing method and sex of the proband. The following parameters were no significant confounders in our data set: intrapersonal variation over one week, cannula, time before and after centrifugation and filling level of collection tubes.

**Conclusion:**

VEGF-A measurement results can be affected significantly by the identified pre-analytical parameters. We recommend the use of CTAD anticoagulant, a standardized type of centrifuge and one central laboratory using the same analyzing method for all samples.

## Introduction

Vascular endothelial growth factor-A (VEGF-A) is a key player in physiologic as well as pathologic angiogenesis [1, 2]. A variety of diseases ranging from tumor growth [3, 4], through asthma [5] to exudative age-related macular degeneration [6] are associated with a deregulation of this angiogenic factor. As a consequence, anti-VEGF therapeutics have been developed to target pathologic angiogenesis in various contexts: Anti-VEGF drugs alone or in combination improved survival rates or progression-free survival rate in certain cancer types [3, 7–11]. Anti-VEGF drugs also have been investigated in ophthalmology in randomized clinical trials [12–15] and have been found to improve treatment outcomes [16, 17].

Along with the increasing clinical role of anti-VEGF compounds, VEGF-A itself has been investigated intensively as a potential diagnostic biomarker or as a predictive marker for treatment response [18–20]. In addition, antagonizing VEGF-A has been found in some studies to be associated with a significantly increased risk of myocardial infarction, hypertension and stroke [21–23]. Even small amounts of intravitreally injected VEGF-binding agents were found to alter systemic VEGF-A levels [24–26]. As a consequence, determination of systemic VEGF-A levels is part of the systemic safety analysis in various studies investigating VEGF-binding proteins [25, 27–29]. However, due to the use of different protocols in quantifying systemic VEGF-A levels, comparison of the results remains difficult.

The aim of this study was therefore to investigate the independent impact of various pre-analytical parameters on the assessment of VEGF-A levels in blood samples from healthy volunteers. Identifying these sources of bias and noise will improve the reliability of future VEGF-A readings and will help to clarify the role of angiogenic factors in disease and response to therapy.

## Methods

### Sample acquisition and distribution

Blood samples from six healthy volunteers (three male, three female, aged between 20 and 40 years) were taken for the initial data set at three centers, two at each center (one male, one female). For additional experiments, blood samples were taken from eight participants (four female and four male). All procedures for obtaining blood samples were standardized following a detailed protocol: blood was first taken from the male participant, then from the female participant in a lying position. Blood sampling had to be scheduled between 7:30 and 10 a.m. Participants had to come to the site in a fasting status with last meal eaten before midnight the previous day and with no extraordinary stress exposure (e.g. excessive sports) the day before. Blood was taken first with a neonatal cannula and then with a butterfly needle. In order to avoid long stasis, the tourniquet was released directly after venous puncture and before blood was collected. The first drops of blood were discarded. After centrifugation of blood samples and before analysis, all aliquots were stored at -20°C and shipped on dry ice if applicable.

**Fig 1:**
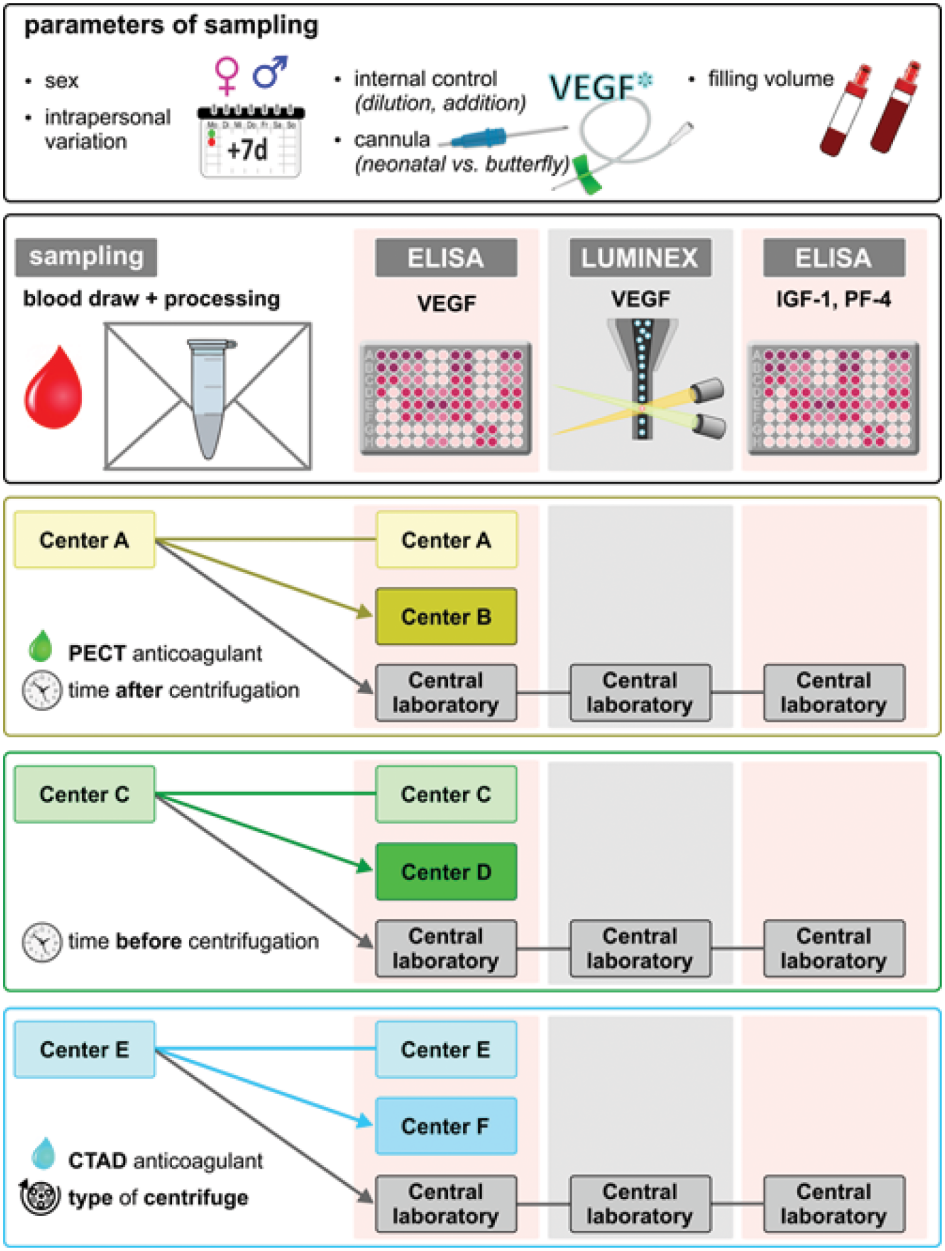
Overview of sample acquisition procedures and the distribution of samples from blood acquisition centers to measuring centers and central laboratory.

Sample acquisition was repeated one week later in order to investigate intrapersonal variations in VEGF-A levels over time. Each center deliberately altered predefined pre-analytical parameters to investigate this parameter's impact on VEGF-A measurements. One aliquot from each sample was measured at the site of blood sampling, one aliquot from the same sample was sent to a second independent measuring center and a third aliquot was sent to a central laboratory. By this approach, each sample was measured independently at three different centers (acquisition center, measuring center and central laboratory) in order to investigate whether the parameter "center" was an independent confounder of VEGF-A measurements. The process of sample acquisition and distribution is illustrated in Fig 1.

### Parameters investigated

All parameters investigated as potential confounders of systemic VEGF-A levels are listed in Table 1.

**Table 1:** Investigated parameters.

Potential confounder	Investigated by
Analyzing center	distribution of identical aliquots to three independent centers for ELISA analysis (using the same batch of ELISA kit R&D Cat No. DVE00)
Anticoagulant	obtaining plasma samples with three different anticoagulants: K_2_EDTA (di-potassium-ethylenediaminetetraacetic acid) **or** PECT (prostaglandin E_1_, EDTA, Na_2_CO_3_ and theophylline) [30, 31] **or** CTAD (citrate, theophylline, adenosine, dipyridamole) [32]). Samples were centrifuged within one hour unless otherwise stated at 3000 g for 15 minutes. EDTA and CTAD samples were kept and processed at room temperature; PECT samples were kept and processed on ice according to Schlingemann et al. [31].
Cannula	using two types of cannulas: 23Gx¾” butterfly cannulas or 23G neonatal cannulas (both from Sarstedt, Nuernbrecht, Germany)
Centrifuge	utilizing two types of centrifuge: fixed-angle rotor (C1) or swing-out rotor (C2) (equivalent settings)
Time before centrifugation	centrifuging several samples (EDTA) immediately (within 20 minutes) after blood withdrawal and leaving other samples at room temperature for two hours; in additional experiments we stored EDTA, PECT and CTAD samples up to 24 hours at their respective storage conditions
Time after centrifugation	storing samples for 3–4 hours vs 6 hours after centrifugation before separation of plasma from cell pellet and subsequent freezing. EDTA samples were kept at room temperature, PECT samples at 4°C.
Intrapersonal variation over time	repeating sampling procedures 7 days later
Filling level	filling a predefined number of Monovette^®^ and Microvette^®^ tubes completely, another set about half
Analyzing method	measuring one aliquot of each batch using Luminex assay (R&D Systems, Human VEGF High Sensitivity Kit; LHSCM293) and one aliquot with ELISA (at the central laboratory)
Thrombocyte activation	measuring platelet-factor-4 (PF-4) using ELISA (R&D Systems, DPF40)
Precision of measurement in very low concentrations	diluting one subset of aliquots 1:10 and one subset 1:5 by adding Dulbecco’s Phosphate Buffered Saline (DPBS)
Recovery of added VEGF-A to human plasma samples	adding freshly reconstituted recombinant human VEGF-A_165_ (R&D Systems; Sf21-derived) to a subset of EDTA, PECT and CTAD samples covering a range of 67 pg/ml to 1,333 pg/ml
Specificity of confounding parameter	measuring IGF-1 (insulin like growth factor-1) using ELISA (R&D Systems; DG100) as an independent biomarker

### Analyzing kits for measured parameters

The measurement of VEGF-A was done at all centers with the same batch of ELISA kit (R&D Cat No. DVE00). At the central laboratory VEGF-A was additionally measured with a Luminex assay (R&D Systems, Human VEGF High Sensitivity Kit Cat No. LHSCM293). Platelet-factor-4 (PF-4) was measured using an ELISA kit from R&D Systems (Cat. No. DPF40). IGF-1 was measured using an ELISA kit from R&D Systems (Cat. No. DG100).

### Missing values and values under the limit of detection

Values that were missing from one center because this center did not have this sample were omitted. Paired analysis was not performed in those cases and samples were excluded from the linear regression model. In total, 6 out of 404 aliquots were excluded due to missing values. Values under the limit of detection (provided by the manufacturer of each kit as minimal detectable dose (MDD)) were uniformly replaced by half the MDD value. For the VEGF-A ELISA assay, MDD was given as 9 pg/ml by the manufacturer, for the VEGF-A Luminex assay MDD was 1,74 pg/ml, for the IGF-1 ELISA MDD was 22,4 ng/ml and for the PF-4 ELISA MDD was 4,63 pg/ml.

### Statistical Analysis

Multiple regression analysis was performed using a linear regression model to assess the independent parameters affecting VEGF-A plasma measurements. Computations were performed using R. Significance of statistical difference is indicated by asterisks *p<0.05, **p<0.01, ***p<0.001; ns: not statistically significant. In addition to the multiple regression analysis we performed descriptive paired analyses using Spearman’s correlation to measure coefficients of determination (R^2^) where appropriate. Data are presented as Tukey box plots and bar charts with standard deviation.

### Ethics

This study was approved by the Ethics Committee at the University of Freiburg, Germany and all participating centers. Participants provided their written informed consent to participate in this study.

## Results

All VEGF-A levels reported in this section were measured by ELISA unless otherwise stated. The central laboratory samples were measured in single aliquots. At all other centers, samples were measured as technical duplicates and mean values were used for further statistical analysis.

### Multiple regression analysis

Results from the multiple regression analysis are shown in Table 2. The following parameters were identified as statistically significant independent confounders of VEGF-A levels: *analyzing center*, *anticoagulant*, *type of centrifuge*, *measuring method* and *sex of the healthy volunteer*. The linear regression model estimates VEGF-A levels measured at the various centers to be between 32 pg/ml lower (center D) and 35 pg/ml higher (center E) compared to the central laboratory that was set as reference. Multiplex bead array measurements are estimated to be on average 38 pg/ml lower compared to ELISA measurements. Female participants had on average 24 pg/ml higher VEGF-A levels compared to male participants in our data set. Both, PECT anticoagulant (-34 pg/ml) as well as in particular CTAD anticoagulant (-73 pg/ml) resulted in significantly lower VEGF-A measurements compared to EDTA. Using a centrifuge with a swing-out rotor yielded on average of 23 pg/ml lower VEGF-A values compared to a fixed-angle centrifuge.

**Table 2:** Results of the multiple regression analysis.

Potential confounder		Mean deviation of VEGF-A results (estimate) [pg/ml] (±SD)	p- values
	(Intercept)	68.1 (±7.1)	<0.001
Analyzing center	Center A vs. **central laboratory**	-5.5 (±6.5)	0.396
	Center B vs. **central laboratory**	-21.3 (±6.6)	0.001
	Center C vs. **central laboratory**	-26.1 (±8.5)	0.002
	Center D vs. **central laboratory**	-32.2 (±8.5)	<0.001
	Center E vs. **central laboratory**	35.2 (±8.0)	<0.001
	Center F vs. **central laboratory**	32.9 (±8.0)	<0.001
Anticoagulant	PECT vs. **EDTA**	-33.8 (±4.8)	<0.001
	CTAD vs. **EDTA**	-73.4 (±7.7)	<0.001
Cannula	neonatal vs. **butterfly**	5.0 (±5.0)	0.322
Type of centrifuge	swing-out rotor vs. **fixed-angle rotor**	-23.2 (±5.7)	<0.001
Time before centrifugation	Time to centrifugation: 2h vs. **30min**	-8.3 (±6.3)	0.188
Time after centrifugation	Time after centrifugation: 3-4h vs. **immediately**	-0.04 (±6.9)	0.995
	Time after centrifugation: 6h vs. **immediately**	7.7 (±7.3)	0.293
Intrapersonal variation over time	week 2 vs. **week 1**	-2.9 (±3.6)	0.423
Sex	Female vs. **male** participant	23.6 (±3.7)	<0.001
Filling level	half filled vs. **completely filled**	7.1 (±4.0)	0.0741
Measuring method	Multiplex bead array vs. **ELISA**	-38.1 (±5.1)	<0.001

Results of the multiple regression analysis; the first two columns show the investigated parameters, whereby the bold printed one was used as reference for this parameter. The column “Mean deviation of VEGF-A results (estimate)” shows the strength of the influence of this parameter in the regression model (± standard deviation)

The following parameters were NOT identified as independent confounders of VEGF-A levels in our data set: *intrapersonal variation over one week* (-3 pg/ml at second vs. first time point), *cannula* (+5 pg/ml for neonatal vs. butterfly), *time before centrifugation* (-8 pg/ml if waiting up to 2 hours), *time after centrifugation* (+8 pg/ml if waiting up to 6 hours) and *filling level of collection tube* (+7 pg/ml for half-filled vs. completely filled tubes).

### Paired analysis

In addition to the linear regression model, we analyzed the following parameters in a descriptive analysis in order to better visualize their impact on mean values and range of measured values.

#### Monovariante center comparison

The box-plot analysis and paired plot comparisons shown in Fig 2.1(a–c) clearly illustrate the variability of results obtained at different centers by measuring aliquots that were taken from the same participants, processed in the same manner and analyzed using the same batch of an ELISA kit. In some cases, a good correlation between the centers and the central laboratory is observed, e.g. center E and F (R^2^ = 0.98 and 0.95, respectively). In contrast, high deviations were found for other centers, for example when comparing center A (R^2^ = 0.79) and center C (R^2^ = 0.5) to the central laboratory. Fig 2.1a depicts results for all samples that were taken at center A with aliquots being sent to center B and the central laboratory for ELISA measurements. It is obvious that measured VEGF-A values from identical aliquots differed depending on the center at which the measurements were performed. A similar effect was observed for samples that were collected at center C with identical aliquots measured at centers C, D and the central laboratory (Fig 2.1b). Fig 2.1c shows good correlation between samples obtained at center E and measured at center E, F and the central laboratory.

**Fig 2:**
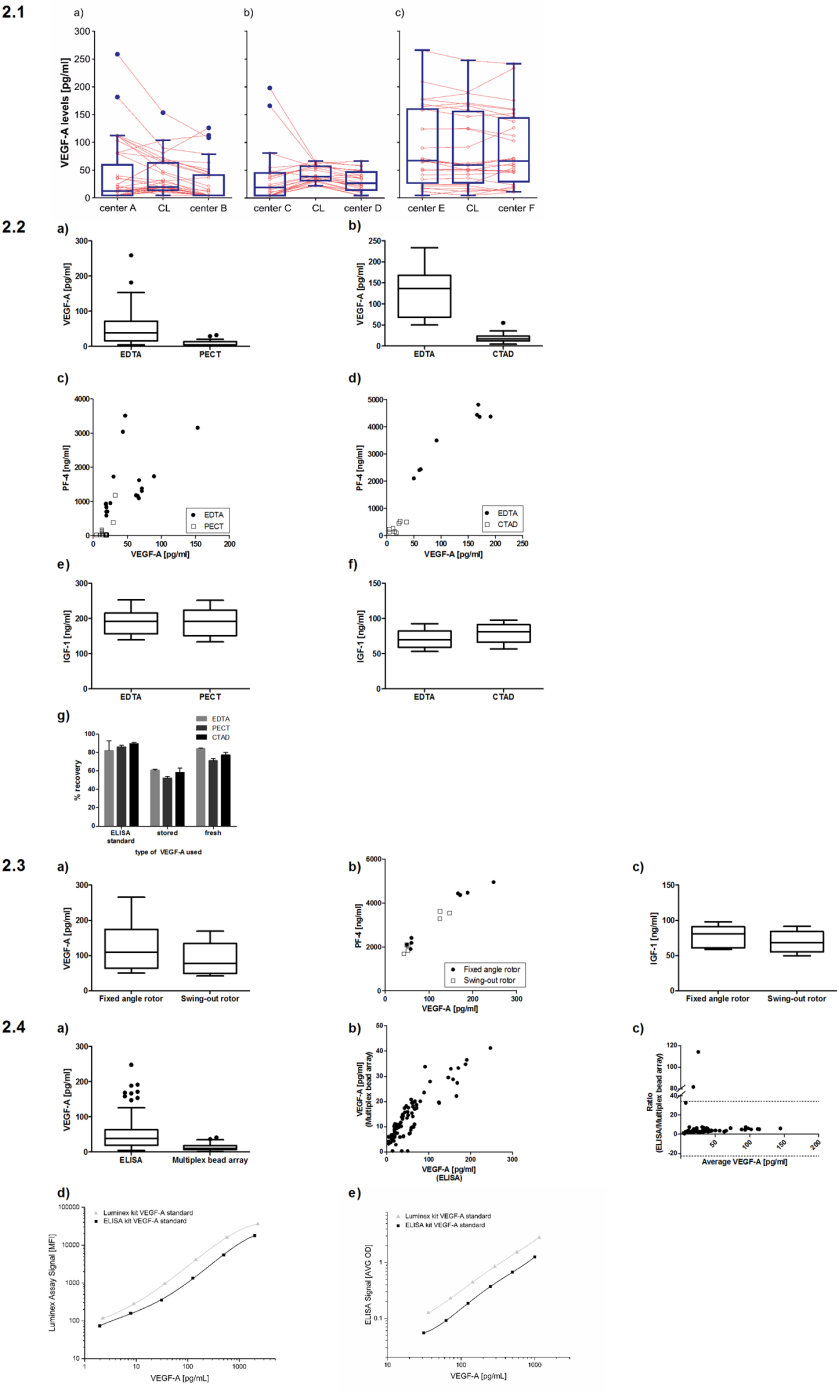
**2.1 (a-c) Paired plots (light orange) and box plots (blue) of VEGF-A levels measured at blood taking centers, measuring centers and at the central laboratory (CL). Connecting lines illustrate the measured VEGF-A levels from identical aliquots of samples measured at the acquisition center, the measuring center and the central laboratory; 2.2 Impact of the anticoagulant (EDTA, PECT, CTAD) on measurement of VEGF-A, PF-4 and IGF-1 from plasma.** VEGF-A levels measured from PECT (a) and CTAD (b) plasma are much lower than from EDTA plasma. Similar to VEGF-A, PF-4 values are higher in EDTA plasma than in PECT (c) or CTAD (d) plasma, indicating increased thrombolysis in EDTA samples compared to PECT or CTAD. IGF-1 levels are not affected by the choice of anticoagulant (e, f) indicating that the choice of coagulant is not a general confounder for all biomarkers. (g) VEGF-A levels obtained after spiking VEGF-A into PBS containing EDTA, PECT or CTAD yielded comparable results thus confirming that CTAD or PECT do not interfere with the VEGF ELISA assay. It was noted, however, that VEGF stored at -80°C for over one year yields lower recovery rates in all samples, irrespective of the anticagulant used; **2.3 Impact of the kind of centrifuge used for separating plasma from the cell pellet (equivalent settings were applied).** VEGF-A (a) and PF-4 (b) levels were higher in some samples from the fixed angle rotor compared to the swing-out rotor. IGF-1 levels (c) were not affected by the type of centrifuge; **2.4 Comparison of measurements obtained with two methods: ELISA vs. multiplex bead array (Luminex).** (a) box-plot analysis showing that absolute values differ between the two measurement methods; (b, c) scatter plot and Bland-Altmann plot, however, demonstrate a good correlation of *relative* measurement results from the two methods; (d, e) VEGF-A protein standards from both the ELISA kit and the Luminex kit were measured in both assays. In either assay, the VEGF-A standard provided with the Luminex kit resulted in higher signals for identical protein concentrations compared to the VEGF-A standard provided with the ELISA kit. This translates in lower measurements of patient sample VEGF-A levels when these are normalized to the Luminex VEGF-A standard as opposed to the ELISA standard (irrespective of the assay used).

#### Anticoagulants: EDTA, PECT and CTAD

VEGF-A in the systemic circulation is stored in thrombocytes to a great extent [33]. Thrombolysis therefore represents a potent confounder for measurement of free VEGF-A plasma levels and must consequently be avoided by using appropriate anticoagulants. EDTA is the most widely used anticoagulant in clinical routine, but both PECT and CTAD anticoagulants have been suggested as alternatives with potential superior effects in preventing thrombocyte activation [30–32]. For both PECT and CTAD, lower amounts of free VEGF-A were measured compared to EDTA plasma in samples from the same participants taken at the same time point using the same method (Fig 2.2a and 2.2b). This may in part be explained by higher thrombocyte activation in EDTA samples compared to CTAD or PECT samples as reflected by higher readings for PF-4, a factor usually stored in thrombocytes and released upon thrombocyte activation (Fig 2.2c and 2.2d). When put into relation to values of VEGF-A levels obtained with EDTA, VEGF-A levels obtained with CTAD were lower than those obtained with PECT, indicating that unwanted release of VEGF-A by thrombocytes may even be lower when CTAD is used compared to PECT. There is no general effect of the anticoagulant on protein biomarkers because IGF-1 determination was not affected by the choice of anticoagulant (Fig 2.2e and 2.2f).

In order to evaluate whether the lower VEGF levels in the CTAD and PECT samples could be due to the two anticoagulants interfering with the ELISA assay, we added known concentrations of recombinant VEGF-A to PBS containing either EDTA, PECT or CTAD (Fig 2.2g). There was no difference in the recovery rate of spiked-in VEGF-A, confirming that CTAD and PECT do not interfere with the VEGF ELISA assay. However, it was notable that older aliquots of recombinant VEGF-A (stored over one year at -80°C) yields significantly lower recovery rates in all samples tested. Furthermore, it was confirmed that both assays measure only free VEGF-A (S1 Fig).

#### Type of centrifuge: fixed angle vs. swing-out rotor

The type of centrifuge could be an important pre-analytic parameter with impact on VEGF-A readings since pellet formation and pellet density in fixed angle vs. swing-out rotor centrifuges may lead to different degrees of contamination of plasma samples with pellet components such as cell debris containing VEGF-A from intracellular sources. In direct comparison, slightly higher VEGF-A levels were detected when samples were centrifuged with a fixed angle vs. a swing-out rotor centrifuge (Fig 2.3a). This difference was identified as an independent confounder of VEGF-A readings in the linear regression analysis (see Table 2). Interestingly, high PF-4 levels, indicative of thrombocyte activation, were mainly observed in samples centrifuged with a fixed-angle rotor (Fig 2.3b). IGF-1 levels were measured as an additional independent biomarker but were not affected by different centrifugation conditions (Fig 2.3c).

#### Measuring method

The most widely used methods to measure VEGF-A are ELISA and multiplex bead arrays [34–38]. Therefore, the amount of VEGF-A was determined in all samples using these two methods. As shown in Fig 2.4, absolute readings from the two assays cannot be compared. In our hands, multiplex bead array measurements resulted in lower values compared to ELISA measurements: Median VEGF-A level measured by ELISA was 36 pg/ml (IQR: 19–63 pg/ml), whereas median VEGF-A level measured by multiplex bead array was 10 pg/ml (IQR: 7–18 pg/ml). This influence of analytical method was identified as an independent confounder in the linear regression analysis (see Table 2). The scatter plot (Fig 2.4b) and the Bland-Altmann plot (Fig 2.4c) indicate a good correlation between the two methods, although absolute values were not identical.

In order to further evaluate the differences between the two measuring methods, we measured standard curves produced with the VEGF calibrator protein supplied with both kits in both assays. The results showed that the VEGF-A provided with the Luminex kit gives higher signals for identical protein concentration (as specified by the manufacturer) irrespective of the assay used. This means that measured sample concentrations, which are back-calculated using a standard curve produced with the calibrator protein from the Luminex kit, result in lower concentrations of target VEGF independent of the assay used (Fig 2.4d and 2.4e).

#### Extreme values

**Fig 3:**
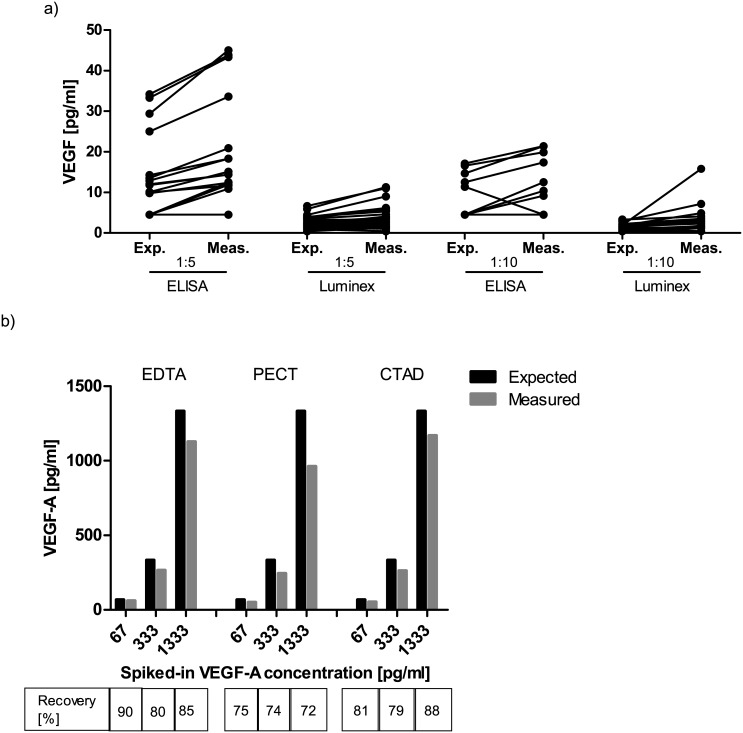
(a) Comparison of the mathematically expected and measured VEGF-A levels after a 1:5 and 1:10 sample dilution. Both ELISA and multiplex bead array measurements measure slightly higher values than mathematically expected after sample dilution; (b) Addition of up to 1.333 pg/ml of recombinant VEGF-A to a plasma sample yields recovery rates of about 80% in all anticoagulants. Exp. = expected value; Meas. = measured value.

The ability of a method to correctly retrieve known values at the upper and lower assay range is an important parameter for ensuring data consistency for samples containing very high or low analyte concentrations. Therefore, samples with known VEGF-A levels were either diluted or spiked with fixed amounts of VEGF-A (spike-in recovery), and it was investigated if the expected levels of VEGF-A could be determined correctly by ELISA or multiplex bead array (Fig 3).

For diluted samples measured with ELISA, we found values close to the expected analyte concentrations, if a previously measured sample was re-measured in a 1:10 dilution. Samples re-measured by ELISA after a 1:5 dilution tended to result in slightly higher values than mathematically expected (Fig 3a). The multiplex bead array method also measured slightly higher values than expected both after a 1:5 as well as after a 1:10 dilution (Fig 3a).

When samples with spiked-in VEGF-A were measured by ELISA, observed recovery rates were approximately 80% (Fig 3b). These experiments were performed only at our central laboratory to test basic assay performance and were therefore not included in the linear regression model.

#### Time before and after centrifugation

**Fig 4:**
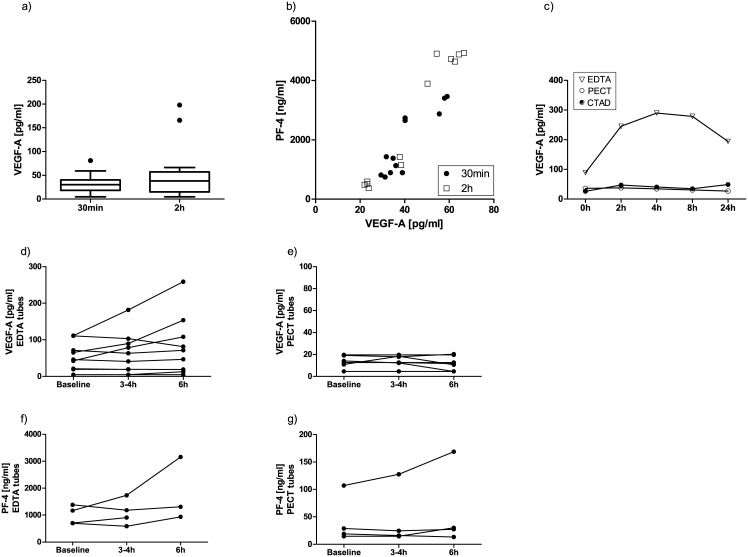
Impact of time before (a,b,c) and after (d-g) centrifugation on measured VEGF-A and PF-4 levels. In some EDTA samples, VEGF-A (a,c,d) and PF-4 levels (b,f) increased with longer incubation times. This was particularly significant when EDTA samples were stored for longer periods of time before centrifugation (c). In PECT samples (c,e,g), VEGF and PF-4 values were not affected by longer sample storage times.

We investigated whether storing blood samples before or after centrifugation would affect VEGF-A readings. When EDTA plasma samples were stored at room temperature for up to two hours before centrifugation, VEGF-A levels were only increased in a few samples (Fig 4a). PF-4 readings also tended to be slightly higher in samples with longer incubation times and correlated with higher VEGF-A readings (Fig 4b). This was, however, not found to be a significant independent confounder of VEGF-A readings in the linear regression model (see Table 2). In an additional experiment, we evaluated whether even longer waiting times (up to 24 hours before centrifugation) would affect VEGF-A readings. While long-term storage before centrifugation had a significant impact on VEGF-A readings from EDTA samples, there was no change in PECT or CTAD samples (Fig 4c).

A subset of EDTA- and PECT plasma samples was stored at room temperature or at 4°C, respectively, for up to 6 hours after centrifugation before the plasma was separated from the cell pellet. In some of the EDTA plasma samples, higher VEGF-A levels were measured (Fig 4d). This was, however, not significant in the linear regression model (see Table 2). Importantly, none of the samples collected in PECT anticoagulant showed a detectable change in measured VEGF-A levels over time (Fig 4e). In support of the notion that PECT anticoagulant very potently inhibits thrombocyte activation, we found considerably higher PF-4 values in EDTA samples compared to PECT samples (Fig 4f and 4g; note the different y-axis in the two PF-4 graphs).

#### Butterfly vs. neonatal cannula

**Fig 5:**
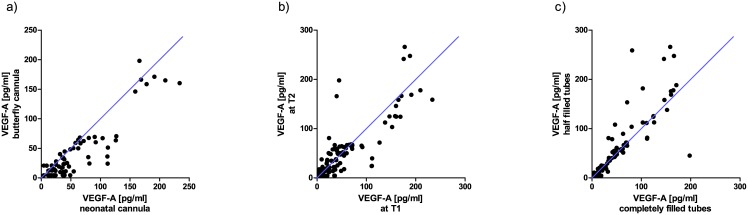
(a) Scatter plot for the influence of cannula (neonatal vs. butterfly) on measured VEGF-A plasma levels; (b) Scatter plot for intrapersonal fluctuations in plasma VEGF-A levels over one week; (c) Scatter plot for the impact of filling level of blood collection tubes on the resulting VEGF-A levels. Location of values near the bisecting line indicates comparable measurement results.

Variations in blood sample acquisition can significantly affect measurement results. For example, this is well established for potassium sampling when release of intracellular potassium leads to erroneous high plasma readings after partial cell lysis during sample acquisition [39]. Likewise, thrombolysis occurring during sample acquisition might result in incorrect high VEGF-A levels. Since VEGF-A measurements have become increasingly important also in neonatal patients with the advent of anti-VEGF treatment in infants suffering from retinopathy of prematurity (ROP), VEGF-A levels were determined in samples obtained with neonatal cannulas or with standard butterfly needles (all taken from the same adult participants). The scatter plot in Fig 5a indicates a minor trend for higher VEGF-A readings in some samples that were obtained with neonatal cannulas. Many values, however, are located near the bisecting line indicating comparable measurement results. The kind of cannula was not a statistically significant independent confounder in the linear regression analysis (see Table 2).

#### Intrapersonal fluctuations over one week

In order to investigate whether VEGF-A levels vary in healthy participants over time, systemic VEGF-A levels were re-measured in new samples obtained from the same individuals after one week. Slight variations were observed, but these were not identified as independent confounders in the linear regression model (Fig 5b and Table 2).

#### Filling level of the collection tubes

In clinical routine, sample tubes are not always filled completely. This leads to altered ratios of sample volume to anticoagulant. Fig 5c shows the scatter plot for completely filled vs. half-filled tubes. There was no difference found (most values are located near the bisecting line) and accordingly, filling level was not identified as an independent confounder in the linear regression analysis (see Table 2).

## Discussion

Systemic VEGF-A levels are measured as biomarkers in different medical fields, such as oncology [30, 40], pneumology [41] and ophthalmology [28, 35–37]. Absolute values for VEGF-A levels vary noticeably between studies (e.g. plasma VEGF-A levels of healthy probands were reported to be between 81 pg/ml [42] and 180 pg/ml [35]) and therefore, cross-study comparisons are difficult. This is due to the fact that the methodology for VEGF-A measurements from human blood differs and that various confounders, e.g. source (plasma or serum [30, 31, 36, 43]), blood sampling techniques and anticoagulants (heparin plasma [44], CTAD plasma [28], EDTA plasma [45] or others [31]), can considerably affect measurable VEGF-A levels. In this study, we identified a number of parameters that can all independently lead to a bias of measured VEGF-A values from human blood samples. These parameters are: type of anticoagulant, assay method, center performing the measurement, type of centrifuge, sex of the participant. These parameters are extremely important to consider when systemic VEGF-A is measured for example as part of a safety evaluation after anti-VEGF therapy or in other fields, where systemic VEGF-A levels are of importance for example in evaluating clinical outcome [18–20] or in providing risk estimates [46, 47].

It is important to emphasize that for this type of study the number of individual probands is not the determining factor (we had only 12 probands in our study providing the blood samples). It is rather the number of *aliquots* retrieved from these samples that determines the validity of such a study. For this purpose, we analyzed a total of 476 aliquots that were all processed in an exactly predefined manner, standardizing all pre-analytic steps but the one which was to be investigated. This allowed us to investigate the individual pre-analytical variable's impact on VEGF-A measurements independent from any inter-individual variations in VEGF-A levels.

There are various explanations why certain pre-analytic steps can affect VEGF-A measurements. For example, it can be speculated that individual factors play a certain role as reflected by the parameter “measurement center”. In our study, all centers used the same batch of ELISA kits, so lot variability can be ruled out as an interfering parameter in our study. Beyond the human factor, different types of spectrophotometers for ELISA read-out may play a role. We therefore recommend using one central laboratory experienced in the applied measurement method.

In order to investigate whether the type of assay applied for analysis has an independent effect on VEGF-A level measurements, all samples were analyzed by multiplex bead array in addition to ELISA. We detected significantly lower VEGF-A values using the multiplex bead array technique compared to the ELISA technique. The Bland-Altmann plot in Fig 2.4c, however, displays a horizontal line (except for three outliers). This demonstrates that relative values are comparable, but that the multiplex bead array retrieved consistently lower absolute values at a relatively fixed fraction from ELISA readings in our hands. This phenomenon was described previously for a comparison of ELISA and multiplex bead array for other cytokines, like IL-1β, TNF-α or IFN-γ [48]. The difference in absolute values was detected despite using internal standard curves in both assays. One possible explanation could be that the VEGF-A used for generating the standard curve differs between the two techniques. Indeed, the calibrator protein provided with the VEGF ELISA kit resulted in lower signal levels in both the ELISA and Luminex system compared to the calibrator protein from the Luminex kit (shown in Fig 2.4d and 2.4e). This explains the lower absolute VEGF-A levels measured in the multiplex bead assay. We therefore conclude that both methods can be used for human plasma VEGF-A measurements but that absolute values cannot be compared between the two techniques.

In contrast to “free” recombinant VEGF-A used for generating standard curves, VEGF-A in plasma samples can be bound to proteins and might therefore escape detection. Both assays used in our study measure only free VEGF as confirmed by addition of aflibercept (see S1 Fig).

One very obvious parameter with significant impact on VEGF-A readings is the anticoagulant used for plasma sampling. Since we were interested in measuring free VEGF-A and since it is well established that high levels of VEGF-A are stored in thrombocytes, serum measurements were ruled out for our comparative analysis. However, even between the different plasma anticoagulants, we found significant differences with regard to their potency to reliably suppress thrombocyte activation. As reflected by PF-4 measurements, the highest degree of thrombocyte activation (and therefore the highest risk of plasma being contaminated by VEGF-A released from thrombocytes) was present in the EDTA samples (both in completely filled and half-filled tubes). This is important, since most centers use EDTA as their standard anticoagulant for plasma sampling. However, for reliable measurement of VEGF-A as a biomarker EDTA does not appear to be the ideal plasma anticoagulant. In both PECT and CTAD buffers, considerably less PF-4 was detected which is indicative of less thrombocyte activation. As a consequence, VEGF-A levels measured in PECT and CTAD buffer showed less variability and were significantly lower than values measured from EDTA plasma. These lower VEGF-A levels in PECT and CTAD buffers do more likely reflect the true free plasma VEGF-A levels, while values from EDTA buffers may be higher due to varying degrees of additional VEGF-A released from thrombocytes. In addition, storing of centrifuged samples *before plasma separation* from the pellet did not affect measurement of VEGF-A when PECT was used as anticoagulant. Similarly, sample storage up to 24 hours *before centrifugation* did not result in different VEGF-A readings when CTAD or PECT samples were used, while EDTA samples resulted in a wide variability of measured VEGF-A levels when samples were incubated over 24 hours before centrifugation. While PECT buffer is currently not commercially available, CTAD tubes can be obtained as standard clinical equipment. For these reasons and since our experiments of VEGF-spiking into PBS containing EDTA, PECT or CTAD did not show any interference of any of the anticoagulants with the ELISA assay, we recommend the use of CTAD plasma for VEGF-A measurements, which is in accordance with the observations from Zimmermann et al. [49]

Starlinger et al. [50] explored in their study, among other parameters, the influence of a Venflon^®^ versus a Vacutainer^®^ butterfly cannula on the measured VEGF-A levels and did not find an effect on measured VEGF-A levels, but on PF-4 levels. Since our study was in part conducted in preparation for the clinical study CARE-ROP, a study for investigating the efficacy and safety of different ranibizumab dosages for the treatment of retinopathy of prematurity (NCT02134457), we compared neonatal with butterfly cannulas. Similar to the results from Starlinger et al., we did not find a significant effect of these two types of cannulas (butterfly vs. neonatal) on measured VEGF-A levels. Neonatal cannulas can therefore be used for VEGF-A sampling.

Svendsen et al. [40] observed that measured VEGF-A levels are highly dependent on the applied centrifugation force: the higher the centrifugation force, the lower the measured VEGF-A levels, possibly due to better separation of thrombocytes from plasma. In our study, we compared two types of centrifuges (swing-out rotor versus fixed angle rotor) using equivalent settings. Even with identical centrifugal forces, VEGF-A levels differed significantly between the two types of centrifuges, with values from swing-out rotor centrifuges being lower than values from fixed-angle centrifuges. PF-4 values tended also to be lower in the swing-out rotor centrifuge, indicating that less thrombocyte activation occurs and therefore less contamination of free plasma VEGF-A with VEGF-A released from thrombocytes. Consequently, we recommend using the same type of centrifuge with identical settings for all samples acquired during the course of a study, preferentially a swing-out rotor centrifuge.

Intrapersonal VEGF-A levels did not significantly vary over one week. This is in line with Svendsen et al. [40] who also did not find a variation in VEGF-A levels within two weeks, but found variations over longer time periods. The fact that VEGF-A levels from the same person measured twice within one week were highly reproducible in our study, demonstrates not only the reliability of our sampling and measuring method but further emphasizes how strong the confounding effects of the interfering parameters are. The fact that IGF-1 measurements were not affected by the investigated pre-analytic parameters further confirms that these identified parameters are specific confounders for VEGF-A measurements.

In conclusion, our study identified potent confounders of VEGF-A measurements that need to be standardized in order to obtain reliable and reproducible results in clinical studies. Based on our results we recommend the use of CTAD as anticoagulant, a standardized type of centrifuge where possible and one central laboratory for measurements of free VEGF-A from human plasma. Either an ELISA or multiplex bead array method can be used but absolute values are not interchangeable between these two assays.

## Supporting Information

S1 Complete Data Set Excel Sheet containing the dataset underlying the findings in the manuscript. (XLS)

S1 Fig ELISA and Luminex standard curves with and without addition of aflibercept. Both in the ELISA as well in the Luminex method, addition of aflibercept reduced the levels of measured VEGF, thus confirming that both assays measure only free VEGF.
(TIF)
