# Supplementary figures and images for: Pre-Analytical Parameters Affecting Vascular Endothelial Growth Factor Measurement in Plasma: Identifying Confounders
Source: PLoS One. 2016 Jan 5;11(1):e0145375. doi: 10.1371/journal.pone.0145375 (PMC4711588; doi:10.1371/journal.pone.0145375)

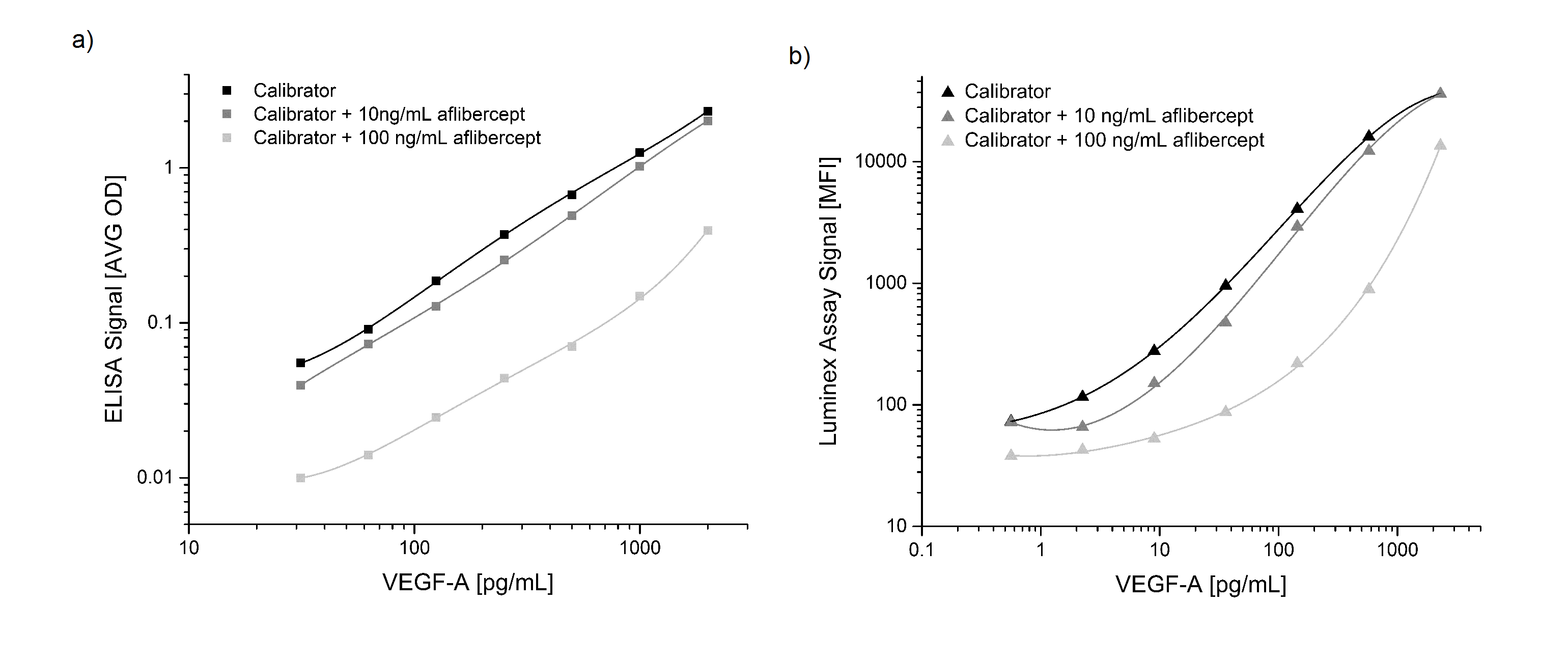

Supplement: S1 Fig — Both in the ELISA as well in the Luminex method, addition of aflibercept reduced the levels of measured VEGF, thus confirming that both assays measure only free VEGF. (TIF) [file pone.0145375.s002.tif]
